# A Ratiometric Fluorescent Probe for Hypochlorite and Lipid Droplets to Monitor Oxidative Stress

**DOI:** 10.3390/bios13060662

**Published:** 2023-06-17

**Authors:** Mousumi Baruah, Anal Jana, Niharika Pareek, Shikha Singh, Animesh Samanta

**Affiliations:** 1 Molecular Sensors and Therapeutics Research Laboratory, Department of Chemistry, School of Natural Sciences, Shiv Nadar (Institute of Eminence Deemed to be) University, Delhi 201314, NCR, India; mb548@snu.edu.in (M.B.); aj228@snu.edu.in (A.J.); np386@snu.edu.in (N.P.); ss336@snu.edu.in (S.S.); 2 Department of Chemistry, University of California, Riverside, CA 92521, USA

**Keywords:** fluorescent probe, ratiometric, ROS, oxidative stress, mitochondria, lysosomes, lipid droplets, viscosity, fluorescence Imaging

## Abstract

Mitochondria are valuable subcellular organelles and play crucial roles in redox signaling in living cells. Substantial evidence proved that mitochondria are one of the critical sources of reactive oxygen species (ROS), and overproduction of ROS accompanies redox imbalance and cell immunity. Among ROS, hydrogen peroxide (H_2_O_2_) is the foremost redox regulator, which reacts with chloride ions in the presence of myeloperoxidase (MPO) to generate another biogenic redox molecule, hypochlorous acid (HOCl). These highly reactive ROS are the primary cause of damage to DNA (deoxyribonucleic acid), RNA (ribonucleic acid), and proteins, leading to various neuronal diseases and cell death. Cellular damage, related cell death, and oxidative stress are also associated with lysosomes which act as recycling units in the cytoplasm. Hence, simultaneous monitoring of multiple organelles using simple molecular probes is an exciting area of research that is yet to be explored. Significant evidence also suggests that oxidative stress induces the accumulation of lipid droplets in cells. Hence, monitoring redox biomolecules in mitochondria and lipid droplets in cells may give a new insight into cell damage, leading to cell death and related disease progressions. Herein, we developed simple hemicyanine-based small molecular probes with a boronic acid trigger. A fluorescent probe **AB** that could efficiently detect mitochondrial ROS, especially HOCl, and viscosity simultaneously. When the **AB** probe released phenylboronic acid after reacting with ROS, the product **AB–OH** exhibited ratiometric emissions depending on excitation. This **AB–OH** nicely translocates to lysosomes and efficiently monitors the lysosomal lipid droplets. Photoluminescence and confocal fluorescence imaging analysis suggest that **AB** and corresponding **AB–OH** molecules are potential chemical probes for studying oxidative stress.

## 1. Introduction

The eukaryotic cells have multiple subcellular organelles such as mitochondria, lysosomes, nuclei, and Golgi bodies which play a crucial role in diverse biological functions in living cells. Among them, mitochondria are considered one of the essential membrane containing cell organelles as they produce energy for the entire body through cellular respiration [1,2]. A substantial amount of reactive oxygen species (ROS) superoxide anion radical (O_2_•^−^) converts into hydrogen peroxide (H_2_O_2_) via the catalytic activity of superoxide dismutase (SOD) [3]. This H_2_O_2_, a common intermediate of ROS, transforms into several potent cytotoxic oxidizing agents and functional biomolecules, such as hypochlorous acid (HOCl), which are generated via a reaction between H_2_O_2_ and chloride ion (Cl^−^) in the presence of myeloperoxidase (MPO) in the cytoplasm [4]. As HOCl is in equilibrium with its conjugate base OCl^−^ (hypochlorite anion) at physiological pH (7.4), it acts as a potent oxidative agent [5]. It also plays a crucial role in the immune system by destroying many pathogens and effecting a few cellular processes, such as proliferation, differentiation, and anti-inflammatory response [6]. However, for a long time, whether HOCl is generated from mitochondria apart from cytoplasm was unclear. Later, Ma et al. proved that HOCl is also produced in mitochondria in macrophage cells [7]. Recently, we demonstrated OCl^−^ generation through NOX2 pathways in cancer cells [8]. Overproduction of OCl^−^ leads to oxidative stress and changes the microviscosity in the cellular organelles [9,10]. Furthermore, the oxidation of mitochondrial fatty acid (FA) upregulates and increases FA accumulation in lipid droplets during oxidative stress [11]. In general, an increased amount of mitochondrial ROS is associated with several diseases, such as chronic kidney disease progression, cancer, diabetes, metabolic disorders, atherosclerosis, and cardiovascular diseases [12,13,14].

Small molecular fluorescent probes and corresponding super-resolution imaging are widely considered one of the most powerful techniques for revealing facts at the molecular level due to their simplicity, high resolution, and low cost [15]. It has become one of the most emerging diagnostic modalities in biomedical research and clinical applications [16,17,18]. Although several small molecular probes are reported to detect an individual analyte, either HOCl or lipid droplets, there are no systematic studies for dual analytes, including OCl^−^ and lipid droplets, during oxidative stress [19]. Therefore, there is an urgent need to develop an efficient probe for monitoring both HOCl and lipid droplets (LDs) during drug-induced oxidative stress conditions. 

A ratiometric fluorescent probe can overcome a few drawbacks compared to turn on/off by self-calibration, and can emerge as an attractive tool in the bio-imaging field [20,21,22,23,24,25,26,27,28,29,30,31,32,33]. However, designing a ratiometric probe and simultaneously targeting two analytes is always challenging [34]. Herein, we developed an efficient mitochondrial targeting hypochlorite-specific small molecular fluorescent probe, **AB**, which can also monitor mitochondrial viscosity. Confocal fluorescence imaging suggests that the hemicyanine based **AB** probe is nicely localized in mitochondria but translocated into lysosomal lipid droplets when the mitochondrial membrane is disrupted during oxidative stress. The phenylboronic triggering motif in **AB** rapidly converts it into **AB–OH** in the presence of hypochlorite. Due to substantial intramolecular charge transfer (ICT) in the basic environment, **AB–OH** exhibited ratiometric emission change and localized to lipid droplets. It showed a good sensitivity towards lipid droplets, the hallmark of cellular stress during oxidative stress. 

## 2. Materials and Methods

### 2.1. General Information

Unless otherwise stated, all the compounds were used as such as they were purchased from commercial vendors without any additional purification. All the substances used in the reactions were bought from Sigma-Aldrich, Alfa Aesar, T.C.I. India, Spectrochem, and Rankem chemicals. Materials used for the spectroscopic and biological analysis are of highest quality available in the brand. Deuterated NMR solvents were purchased from Eurisotope. Dulbecco’s modified Eagle’s medium (DMEM), 10% fetal bovine serum (FBS), and 100× Antimycotic antibiotics were purchased from Thermo Fisher. Silica gel G-60 F254 aluminum TLC was used to monitor reaction completion, and short- and long-wavelength UV lamp chamber was used to visualize the TLC Silica gel with a mesh size of 100–200 was used for column chromatography. Distilled MilliQ water was used wherever water was needed. 

### 2.2. General Instrumentation

NMR spectra of the synthesized molecules were recorded in the Bruker Avance 400 NMR spectrometer, Germany, using deuterated solvents. All the mass spectra were recorded in Agilent 6540, Q-TOF LC/MS system (Agilent Technologies, Santa Clara, CA, USA) connected with Agilent 1290 UPLC using DI water and acetonitrile along with 0.1% TFA as mobile phase with a gradient solvent system. The analytes 4% sodium hypochlorite solution was purchased from Qualigens (cat no. 7681-52-9) and 50% Hydrogen Peroxide solution from Fisher Scientific (cat no. 7722-84-1). Using an Agilent Cary 8454 UV-Vis diode array spectrophotometer, all the absorption spectra were measured, whereas the emission spectra were measured in HORIBA Fluorolog-3 spectrofluorometer (Model: FL3-2-IHR). The scan slits for excitation and emission were adjusted to 2 nm. A Waters Alliance System (Milford, MA, USA) equipped with an e2695 separation module, and a 2998 photodiode-array detector was used for the HPLC experiments. The cells were incubated in a Thermo Fisher CO_2_ incubator, and imaging was performed with Nikon confocal microscope.

### 2.3. Synthesis of AB

0.343 mmol of 2,3-dimethylbenzo[d]thiazol-3-ium (B) and 0.343 mol of (4-((4-formylphenoxy)methyl)phenyl)boronic acid (C) was mixed in dried ethanol in the presence of a catalytic amount of piperidine. The mixture was allowed to stir for 6 h, and the precipitate was filtered and washed with cold diethyl ether to render a 78% yield. The product formed was characterized using HRMS and NMR Spectroscopy. (Detailed schemes for the synthesis and the characterizations are provided in the supplementary material).

### 2.4. Cell Culture Experiments

Adenocarcinoma human cell line A549 was purchased from NCCS Pune. All the cell culturing was performed in DMEM with 10% FBS and 1% Antimycotic under an atmosphere of 5% CO_2_ at 37 °C. The biocompatibility of the probe was determined using the standard MTT [(3-(4,5-Dimethylthiazol-2-yl)-2-5-Diphenyl tetrazolium Bromide] assay. 

Cell imaging experiments were performed in 35mm confocal dishes with A549 cells. Cells were seeded one day before the experiment and treated with AB (10 μM, 30 min), in addition with and without the treatment, of different stress-inducing agents such as zymosan, PMA (Phorbol 12-myristate 13-acetate), MPO (Myeloperoxidase), and different drugs such as doxorubicin and paclitaxel. Images were taken in a confocal microscope.

## 3. Design Principle

To develop the targeted fluorescent probe, we conjugated a suitable trigger for reactive oxygen species (ROS) with a simple hemicyanine type of fluorophore. An aryl boronic acid has been used for developing several fluorescent probes for reactive oxygen species, especially hydrogen peroxide (H_2_O_2_) [35,36,37]. The basic concept was that the boronic acid ester will exhibit a photoinduced electron transfer (PET) mechanism and thus would be non-fluorescent after oxidation, and with the PET is switched off we get a fluorescent signal. A few reports also demonstrated that ONOO^−^ (peroxynitrite) [38,39] and HOCl [40,41] oxidized boronic acid derivatives to phenol, resulting in a turn-on fluorescence response. However, depending on fluorophores and triggers attached to boronic acid or boronic ester (boronic acid pinacol ester), the selectivity differs towards each specific reactive oxygen species. Herein, we also choose simple boronic acid, which has good water solubility and photostability and can be an active intermediate for targeting reactive oxygen species.

**Figure 1:**
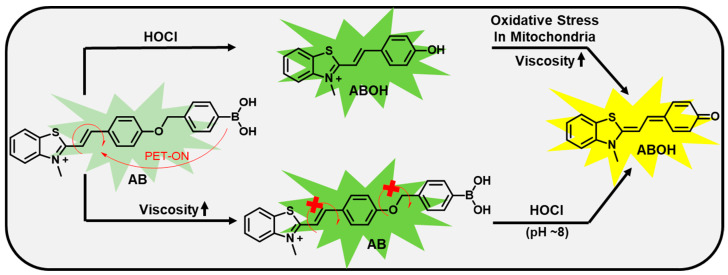
Design principle and sensing mechanism of **AB**. The probe AB is initially weak fluorescent (light green) in aqueous media due to the PET (photoinduced electron transfer) process but shows strong fluorescence in high viscous media (green color). ABOH exhibits stronger emission (represented as green) due to PET off mechanism. In mitochondrial pH (~8.0) and hence ABOH exhibits a longer wavelength (represented as yellow) due to ICT (intramolecular charge transfer) process.

Furthermore, flexible hemicyanine fluorescent probes can respond to viscosity and associated lipid droplets during oxidative stress. Thus, a simple n-methylthiazolium acceptor was conjugated with phenylboronic acid via a Knoevenagel condensation reaction to yield our desired hemicyanine-based probe **AB.** The **AB** probe exhibited a ratiometric fluorescent response through an ICT process (Figure 1) while reacting with ROS, especially hypochlorite. Herein, we reported the development of probe **AB** and its application for dual analytes that include a hypochlorite in mitochondria and lipid droplets in lysosomes during oxidative stress with straightforward synthetic methodology.

## 4. Photophysical Properties

Chang’s [42] and James’s groups [43] reported that boronic acid derivative is specific to H_2_O_2_. Shu et al. synthesized boronic acid derivatives, which showed specificity towards ONOO^−^ with low LOD (16 nM) [44]. However, this probe almost remained silent towards HOCl and reacted slowly with H_2_O_2_. Thus, we first screened our probe with probable reactive oxygen species, including H_2_O_2_, HOCl, OH·, and ONOO^−^ (Figure S1). Interestingly, we found the phenylboronic acid derivative of thiazolium was more specific towards hypochlorite than hydrogen peroxide (H_2_O_2_) and ONOO^−^.

**Figure 2:**
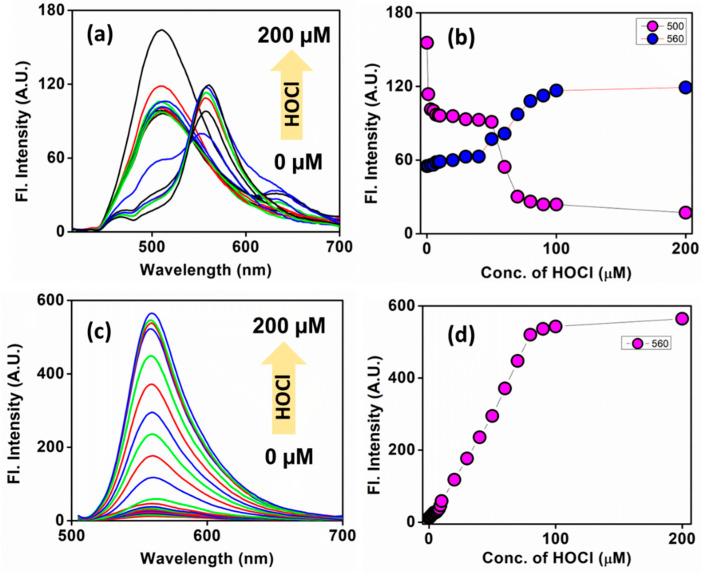
Emission spectra of **AB** in the presence of different concentrations of HOCl (0–200 µM). (**a**) λ_ex_ = 400 nm and (**c**) λ_ex_ = 490 nm and their corresponding scatter plots (**b**,**d**). Probe concentration: 10 µM. The HOCl concentrations used were 100nm, 300 nm, 500 nm, 700 nm, 1–10 µM (10 points), 10–100 µM (10 points), and 200 µM.

Firstly, we carefully checked the sensing ability of the **AB** probe for HOCl in alkaline pH (pH = 8), as the reaction is much more feasible in alkaline conditions (Figure 2). Since H_2_O_2_ is an intermediate for generating HOCl, we studied the photophysics concerning H_2_O_2_. We did a reaction kinetics with H_2_O_2,_ as shown in Figure S2. All three oxygen reactive species (hypochlorous acid, hydrogen peroxide, and peroxynitrite) can cleave the arylboronic acid trigger, which acts as a receptor for photoinduced electron transfer in the buffer. The phenylboronic acid cleaved and formed a phenol derivative that exhibited intramolecular charge transfer (ICT) depending on pH and other microenvironments [45]. For a systematic study, a time-dependent kinetics reaction was recorded by monitoring at 400 nm the maximum absorbance in UV spectra for 80 min after adding 100 µM H_2_O_2_ and HOCl (Figures S2 and S3). The maximum absorbance at 400 nm gradually decreased and the new peak at 490 nm steadily increased with time (Figure S2). In the case of HOCl, the reaction kinetics were much faster than with H_2_O_2_, as the product peak reached a maximum within 15 min (Figure S3). A similar reaction kinetic experiment was conducted using maximum emission at 560 nm, and the reactivity towards HOCl was almost four times faster than H_2_O_2_ (Figure S6).

Next, the ratiometric emission change was analyzed towards variable concentrations of both the analytes (H_2_O_2_ and HOCl) ranging from 0−200 µM. The emission peak at ~500 nm was gradually decreased, and simultaneously a new peak at ~560 slowly appeared with increased concentration while exciting at 400 nm. When the same molecule was excited at 490 nm, a gradual increment at 560 nm with increased H_2_O_2_ and HOCl concentrations was observed. Hence, **AB** can be considered an efficient fluorescent probe for HOCl (Figure 2 and Figure S4) and remains partial towards H_2_O_2_.

Oxidative stress in living cells is associated with the overproduction of reactive oxygen species, including HOCl and H_2_O_2_. When **AB** reacts with these reactive species (HOCl or H_2_O_2_), it releases a quinone methide to form **AB–OH**. This phenomenon was confirmed using HPLC (Figure S7). **AB–OH** exhibited ratiometric emission properties due to the intramolecular charge transfer (ICT) from the donor phenoxide to the n-methylthiazolium acceptor in different pH. Thus, we first checked the absorbance and emission spectra of **AB–OH** in different pH. The absorbance at 400 nm was gradually reduced with increasing pH from 4 to 10. However, a new absorbance peak at 490 nm was gradually increased with pH from 4 to 10. The maximum emission at 510 nm was gradually decreased with increasing pH from 4 to10 upon excitation at 400 nm. However, the emission maxima at 560 nm increased when the pH was increased from 4 to 10 upon excitation at 490 nm. As the ratio of emission intensity at 500 and 560 nm changes, this probe can be considered a potential ratiometric probe toward pH alteration, which is associated with elevated ROS concentrations (Figure S5). We also performed a selectivity study after the addition of different analytes and found it to be reactive with all four ROS, but the best reactivity was observed in the case of HOCl, and thus we did all the further studies considering **AB** as an HOCl probe (Figure S8).

This **AB**/**AB–OH** fluorescent probe may respond to viscosity due to rotatable bonds. The fluorescence intensity at 500 nm significantly increased with an increase in viscosity when excited at around 400 nm (Figure S5). Hence, **AB** can be considered an efficient probe for hypochlorite and viscosity, and the corresponding **AB–OH** could be a viscosity sensor.

## 5. Biological Studies

**Figure 3:**
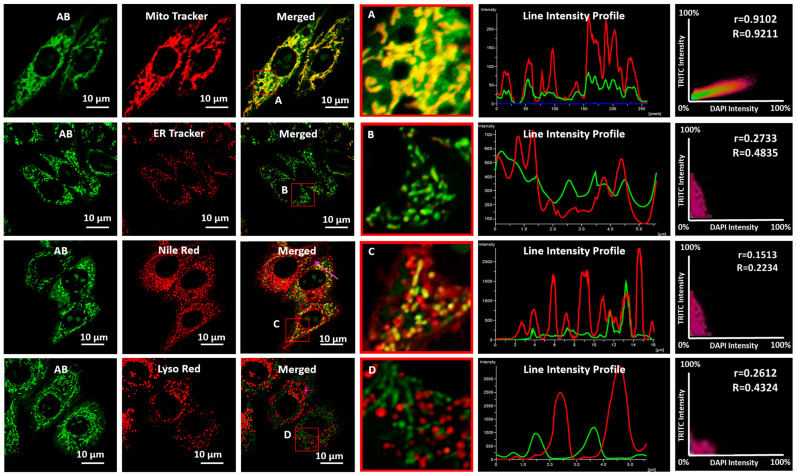
Confocal fluorescence images for co-localization of AB (10 µM) in A549 cells. Images were taken after co-staining with Mito Tracker Deep red, ER Tracker Red, Nile red, and Lyso Tracker Deep red. The squares marked “A”, “B”, “C”, and “D” signify the zoomed-in picture. Corresponding line and scatter plots, wherein “r” and “R” represent Pearson’s correlation and Mander’s overlap coefficients, respectively. Images were captured using a 100× oil emersion lens with 2× zoom. Image scale bar = 10 µm.

Its biocompatibility is the first and foremost criterion for a fluorescent probe to be efficiently used for cellular imaging. To test this, we subjected our probe **AB** to A549 cells for 24 h and found it was almost nontoxic (Figure S9) by MTT assay compared to the 10 µM positive control doxorubicin. A co-localization study confirmed that the probe was nicely localized in mitochondria with a high Pearson’s coefficient (0.9102). There was a partial overlap with the endoplasmic reticulum (ER) but almost no overlap with lipid droplets or lysosomes (Figure 3). The high specificity to mitochondria suggested that the cationic **AB** probe has a great affinity to accumulate in the negative mitochondrial matrix. 

**Figure 4:**
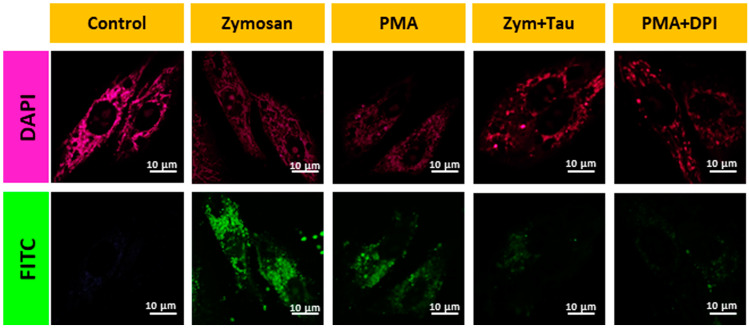
Confocal images for A549 cells during the oxidative stress conditions using inducers Zymosan, PMA, and inhibitors such as taurine and DPI. Image scale = 10 µm.

**Figure 5:**
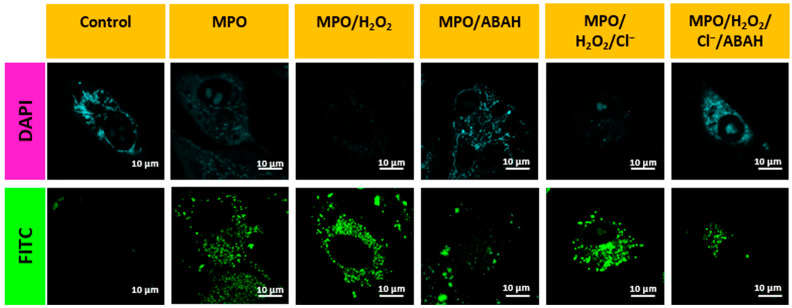
Confocal images for A549 cells during the oxidative stress conditions using inducers MPO H_2_O_2_ and chloride ions, and inhibitor ABAH Image scale = 10 µm.

Although **AB** responded to three reactive oxygen species, the most sensitivity towards hypochlorite encourages us to study intracellular ROS, especially hypochlorite, in living cells. We studied the efficiency of the **AB** probe in detecting endogenous mitochondrial HOCl in live cells. For this, we pretreated the cells with different known endogenous HOCl inducers such as PMA (500 nM, 1 h), Zymosan (10 µg/mL, 1 h), MPO, MPO/H_2_O_2_, MPO/H_2_O_2_/Cl^−^, and then with **AB** for 30 min. Compared to the control, there was an apparent enhancement of fluorescence intensity for all the cases as the amount of HOCl was increased (Figure 4 and Figure 5). In contrast, cells treated with inhibitors such as 4-aminobenzoic acid hydrazine (4-ABAH), taurine, and DPI exhibited very low fluorescence in the FITC channel compared to the non-treated cells (Figure 4 and Figure 5). Therefore, all the above results confirm the AB probe’s proficiency in detecting endogenous HOCl generated via MPO and Zymosan.

**Figure 6:**
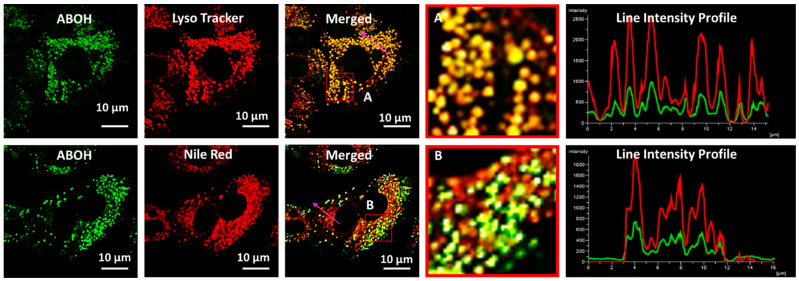
Confocal fluorescence images for co-localization of **AB–OH** (10 µM) in A549 cells. Images were taken after co-staining with Lyso Tracker Deep Red and Nile Red. The squares marked “A” and “B” signify the zoomed-in picture, followed by the corresponding line plots showing the overlap of the probe with commercial trackers. Images were captured using a 100× oil emersion lens with 2× zoom. Image scale bar = 10 µm.

Previously reported boronic acid-based fluorescent probes did not systematically study how the product, phenolic acid derivative (after reaction with ROS) behaves in cells during oxidative stress. Herein, we carefully studied the fate of the fluorescent probe after its response towards ROS using high-resolution confocal fluorescence microscopic images. The **AB–OH**, a product of **AB** during oxidative stress, was localized in the same or other intracellular organelles. As shown in Figure 6, **AB–OH** was nicely localized in lysosomes instead of mitochondria. These results indicate that the **AB** probe is initially localized in mitochondria but translocated to lysosomes when HOCl induced cleavage of phenylboronic acid. The mitochondria are depolarized during oxidative stress, resulting in a lowering of MMP (mitochondrial membrane potential). Thus, the probe leaks out from mitochondria and is localized to lysosomes.

**Figure 7:**
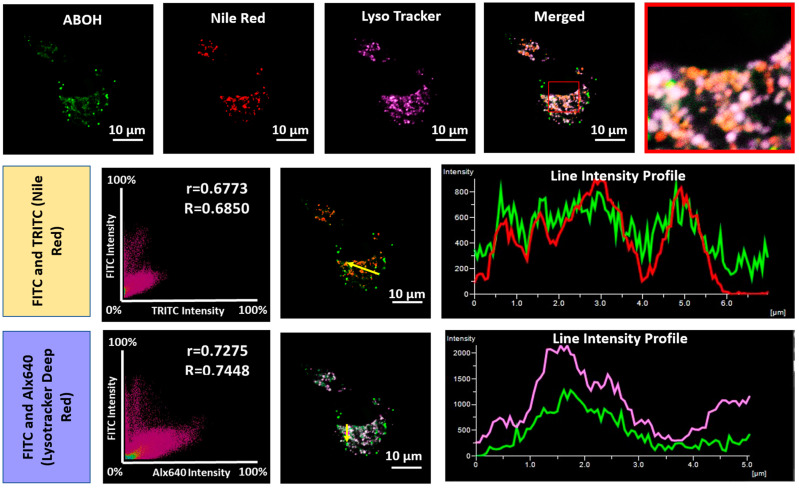
Confocal fluorescence images for co-localization of **AB–OH** (10 µM) in A549 cells to confirm co-localization in lysosomal lipid droplets. After inducing oxidative-stress conditions, images were taken after co-staining with Lyso Tracker Deep Red and Nile Red together. The squares signify the zoomed-in picture. Below are the corresponding line and scatter plots, wherein “r” and “R” represent Pearson’s correlation coefficient and Mander’s overlap coefficient for two channels simultaneously. Images were captured using a 100× oil emersion lens with 2× zoom. Image scale bar = 10 µm.

As spectroscopic analysis of **AB–OH** exhibited an excellent response to viscosity, **AB–OH** can be a potential biomarker for lipid droplets that can monitor the oxidative process. A co-localization experiment suggested that **AB–OH** nicely localized in lysosomal lipid droplets with a Pearson coefficient (0.6773). However, it remains silent to non-lysosomal lipid droplets. As shown in Figure 7, **AB–OH** nicely colocalized with lysosomal LDs, confirmed by the merged images with commercial trackers Lyso Tracker Deep Red and Nile Red, along with the **AB–OH** probe in the FITC channel, respectively. 

**Figure 8:**
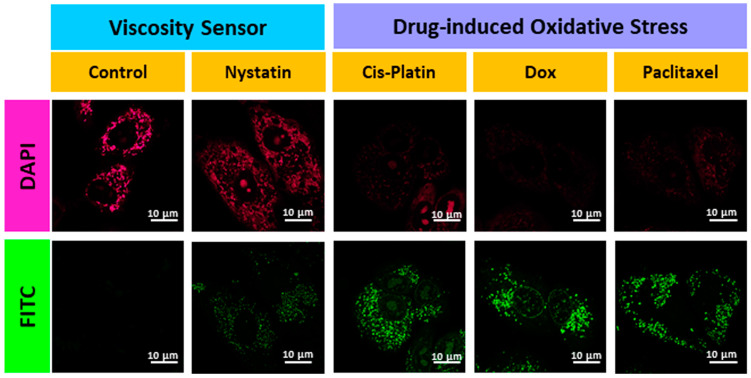
Confocal images for A549 cells during increased mitochondrial viscosity after treatment with nystatin and in oxidative stress conditions using known drugs such as cis-platin, doxorubicin, and paclitaxel. Image scale = 10 µm.

Further, to confirm the mitochondrial viscosity response of **AB**, cells were pretreated with nystatin, which is known to cause swelling of mitochondria, resulting in increased mitochondrial viscosity [46,47,48,49,50,51]. There was an enhancement in fluorescence intensity in the DAPI and FITC channels compared to the control (Figure 8). Finally, to test the drug-induced oxidative stress in cancerous cells, the cells were pretreated with well-known anticancer drugs such as doxorubicin, cisplatin, and paclitaxel for 1 h **AB** was incubated for 30 min before confocal microscopic imaging. A significant enhancement in fluorescence intensity was observed in the FITC channel compared to the untreated cells, and the change in morphology confirmed that the cells were under oxidative stress (Figure 8). This led to mitochondrial damage, consequently, changes in MMP and the formation of lipid droplets. Hence, a simple AB probe can efficiently sense mitochondrial ROS, especially HOCl and lysosomal viscosity in cells under oxidative stress conditions.

## 6. Conclusions

Reactive oxygen species (ROS) play crucial roles in redox signaling in living cells. However, overproduction of these reactive species may damage nucleic acids and oxidize proteins. These adverse effects may lead to cell death, neuronal diseases, cancer, and diabetes. Hence, we studied ROS, especially HOCl, in cancer cells during oxidative stress caused by viral infections and drug treatment. This may alter cellular microenvironments, including cellular viscosity and corresponding lipid droplets. Hence, we systematically studied the detection of hypochlorite in mitochondria during oxidative stress and the post-effect using a simple molecular probe that exhibited ratiometric properties. We successfully synthesized a derivative of hemicyanine dye with boronic acid as the trigger and receptor of photoinduced electron transfer. This **AB** showed good sensitivity towards hypochlorite compared to peroxynitrite (ONOO–) and the most common and highly abundant hydrogen peroxide (H_2_O_2_). It also responded towards viscosity with turn-on property. These excellent photophysical properties offer a new scope for studying confocal fluorescence imaging in living cells to monitor HOCl and viscosity during oxidative stress. **AB** showed a good co-localization in mitochondrial and responded towards HOCl ratiometrically during oxidative stress. We have experimentally verified and proved the significance of our designed probe during a change in mitochondrial viscosity. Confocal image analysis indicated that **AB–OH**, a product of **AB**, translocates to lysosomes and monitors the lysosomal lipid droplets during oxidative stress. Hence, this simple fluorescent probe can be a potential biomarker to study redox imbalance and lipid droplets during oxidative stress.

## Data Availability

Not applicable.

## Supplementary Materials

The following supporting information can be downloaded at: https://www.mdpi.com/article/10.3390/bios13060662/s1.
