# Supplementary material for: A Ratiometric Fluorescent Probe for Hypochlorite and Lipid Droplets to Monitor Oxidative Stress
Source: Biosensors (Basel). 2023 Jun 17;13(6):662. doi: 10.3390/bios13060662 (PMC10295842; doi:10.3390/bios13060662)
Supplement: Supplementary file 1 [file biosensors-13-00662-s001.zip › biosensors-2406285-supplementary.pdf]

A Ratiometric Fluorescent Probe for Hypochlorite and Lipid Droplets to Monitor Oxidative Stress

Mousumi Baruah[†1], Anal Jana[†1,2], Niharika Pareek[1], Shikha Singh[1], and Animesh Samanta*[1]

[1]Molecular Sensors and Therapeutics Research Laboratory, Department of Chemistry, School of Natural Sciences, Shiv Nadar Institute of Eminence (SNIoE) Deemed to be University, Delhi NCR, NH91, Dadri, Gautam Buddha Nagar 201314, India.

E-mail: animesh.samanta@snu.edu.in

[2]Department of Chemistry, University of California, Riverside, California, 92521, United States.

†Equal Contribution

1. Synthetic Strategy

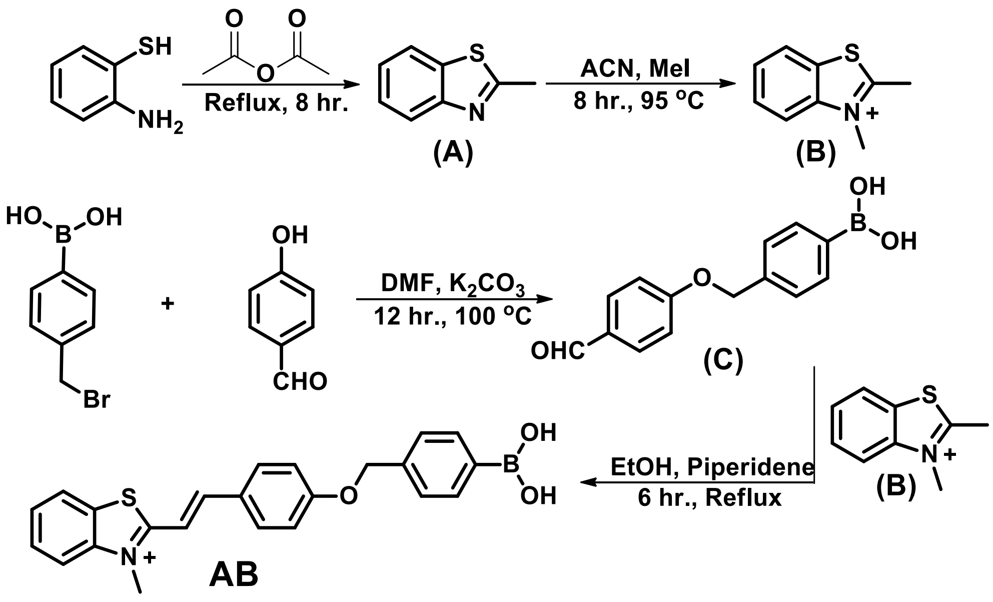

Scheme S1: Scheme for the synthesis of AB.

Synthesis of 2-methylbenzo[d]thiazole (A) An oven-dried round-bottomed flask was charged with 10 ml of acetic anhydride and 500 μL of 2-amino thiophenol. The mixture was allowed to stir for 8 h, and the reaction was continuously monitored with TLC. Upon completion of the reaction, the mixture was allowed to cool, and the solvent was removed in a vacuum. After removing the solvents, 50 mL of water was added and neutralized with NaOH solution. The product was extracted in ethyl acetate and further purified with column chromatography. The compound (A) was obtained as a yellow liquid (58% yield). ^{1}H NMR (400 MHz, DMSO) δ 8.03 (d, J = 7.9 Hz, 1H), 7.91 (d, J = 8.2 Hz, 1H), 7.50 – 7.45 (m, 1H), 7.39 (t, J = 7.6 Hz, 1H), 2.80 (s, 3H). ^{13}C NMR (101 MHz, DMSO) δ 167.02, 152.96, 135.19, 125.99, 124.72, 121.96, 121.93, 19.75.

Synthesis of 2,3-dimethylbenzo[d]thiazol-3-ium (B) 500 mg of (A) was dissolved in 2 ml dried ACN and taken in and mixed with an excess amount of methyl iodide. The mixture is heated at 95 ^{o}C for a period of 8 h. The tube was allowed to cool to room temperature, and 2 ml diethyl ether was added to the reaction mixture. The precipitate obtained was filtered and washed with cold diethyl ether. The product was obtained as a white solid with a 67% yield. ^{1}H NMR (400 MHz, DMSO) δ 8.44 (d, J = 8.1 Hz, 1H), 8.29 (d, J = 8.5 Hz, 1H), 7.90 (t, J = 7.9 Hz, 1H), 7.81 (t, J = 7.7 Hz, 1H), 4.20 (s, 3H), 3.17 (s, 3H). ^{13}C NMR (101 MHz, DMSO) δ 177.26, 141.58, 129.25, 128.70, 128.08, 124.50, 116.77, 36.21, 17.14.

Synthesis of (4-((4-formylphenoxy)methyl)phenyl)boronic acid (C) 0.48 mmol of 4 hydroxybenzaldehydes and 0.48 mmol of (4-(bromomethyl)phenyl)boronic acid were mixed in dried DMF with 2 equivalent of K_2CO_3. The mixture was allowed to stir for 12 h at 100°C and poured into cold water after the completion of the reaction. The mixture was neutralized with dil. HCl was extracted in ethyl acetate, and purified products were obtained as a white precipitate by adding diethyl ether (61% yield). ^{1}H NMR (400 MHz, DMSO) δ 9.86 (s, 1H), 8.07 (s, 2H), 7.87 (d, J = 8.8 Hz, 2H), 7.81 (d, J = 8.0 Hz, 2H), 7.42 (d, J = 8.0 Hz, 2H), 7.20 (d, J = 8.7 Hz, 2H), 5.24 (s, 2H). ^{13}C NMR (101 MHz, DMSO) δ 191.33, 163.29, 138.04, 134.29, 131.82, 129.78, 126.70, 115.32, 69.65.

Synthesis of (E)-2-(4-((4-boronobenzyl)oxy)styryl)-3-methylbenzo[d]thiazol-3-ium (AB) 0.343 mmol of (B) and 0.343 mol of (C) was mixed in dried ethanol in the presence of a catalytic amount of piperidine. The mixture was allowed to stir for 6 h, and the precipitate obtained was filtered and washed with cold diethyl ether (78% yield). ^{1}H NMR (400 MHz, DMSO) δ 8.41 (d, J = 7.8 Hz, 1H), 8.20 (t, J = 12.3 Hz, 2H), 8.06 (d, J = 10.2 Hz, 3H), 7.96 – 7.73 (m, 5H), 7.44 (d, J = 8.0 Hz, 2H), 7.22 (d, J = 8.8 Hz, 2H), 5.26 (s, 2H), 4.33 (s, 3H). ^{13}C NMR (101 MHz, DMSO) δ 172.05, 161.96, 148.58, 141.96, 138.07, 134.21, 132.04, 129.22, 128.18, 127.53, 126.96, 126.65, 124.10, 116.63, 115.63, 111.33, 69.62, 36.16.

Synthesis of (E)-2-(4-hydroxystyryl)-3-methylbenzo[d]thiazol-3-ium (AB-OH)

AB-OH was synthesized to get a better insight into spectral properties and colocalization of the product thus formed after the reaction of AB with ROS

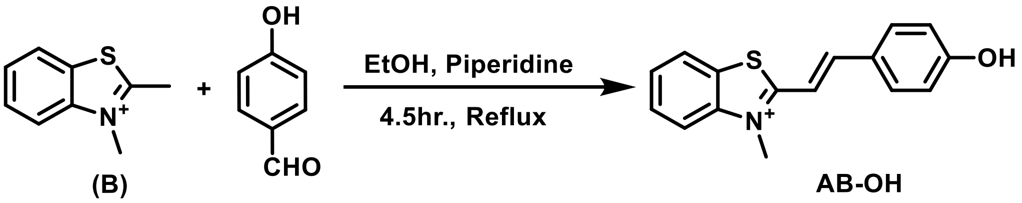

Scheme S2: Scheme for the synthesis of AB-OH.

0.343 mmol of (B) and 0.343 mmol of 4 hydroxybenzaldehyde were mixed in ethanol and cat. amount of piperidine was added. The reaction mixture was allowed to stir for 4.5h, and the solid product obtained was washed with diethyl ether. ^{1}H NMR (400 MHz, DMSO) δ 10.61 (s, 1H), 8.39 (d, J = 7.7 Hz, 1H), 8.20 (d, J = 8.4 Hz, 1H), 8.14 (d, J = 15.7 Hz, 1H), 7.96 (d, J = 8.7 Hz, 2H), 7.88 – 7.73 (m, 3H), 6.93 (d, J = 8.7 Hz, 2H), 4.31 (s, 3H). ^{13}C NMR (101 MHz,

DMSO) δ 172.10, 162.16, 149.26, 141.99, 132.57, 129.18, 128.07, 127.40, 125.38, 124.10, 116.57, 116.31, 110.08, 39.52, 36.12.

2. MTT Assay

Approximately 10000 cells were incubated in 96-well plates for 24 h, after which the adherent cells were treated with the necessary doses of AB, and washed after 24 h. After incubation, 10% final concentration of MTT solutions were added (as provided by Hi-Media Laboratory, Mumbai, India), and incubation was continued for 4 h while a Leica microscope was used to continuously examine the needle-shaped crystal appearance. Following careful removal of the incubation media, 100 μL of DMSO was added, and was further incubated for an additional 10 minutes to complete the crystal dissolution. Following that, absorbance at 570 nm and the percentage of cell viability was calculated using the formula:

$$\text{Viability (\%)} = \text{Abs. sample/Abs. control} \times 100$$

3. Reagent Preparation for selectivity study.

<u>$OH^{\cdot}$ radical:</u> Fenton chemistry concept was used to generate hydroxyl radical. A ferrous chloride (1 eq.) solution and a solution of H_2O_2 (10 eq.) were mixed together. The concentration of $OH^{\cdot}$ was equivalent to the Fe (II) concentration (10 mM).

<u>$ONOO^-$:</u> Peroxynitrite solution was synthesized according to the literature report. (Robinson, K. M., and Beckman, J. S. (2005) *Methods Enzymol*. 396, 207−214). In summary, sodium nitrite (0.6 M), hydrogen peroxide (0.6 M), hydrochloric acid (0.7 M), and sodium hydroxide (3 M) were prepared. The solutions 0.7 M HCl + 0.6 M H_2O_2 and 0.6 M sodium nitrite were loaded into individual syringes connected with a “T” connector. Contents of the individual syringes were mixed with the help of a syringe pump running at a flow rate of 17 mL/min. After mixing, the reaction was quenched by collecting the eluent in a beaker containing 3 M NaOH on ice. The resulting solution was split into small aliquots and stored at -80 ^{o}C. The aliquots were thawed immediately before use, and the concentration of peroxynitrite was determined by measuring the absorption of the solution at 302 nm.

All the other reagents were used as it is by preparing a stock of 100mM concentration.

4. Selectivity study with different ROS

The selectivity study of AB towards different ROS was done in PBS buffer (pH 7.4). The spectra were recorded immediately after adding different ROS to check the change in

fluorescence intensity compared to the control AB. This confirmed the fast reactivity of HOCl over all the other ROS.

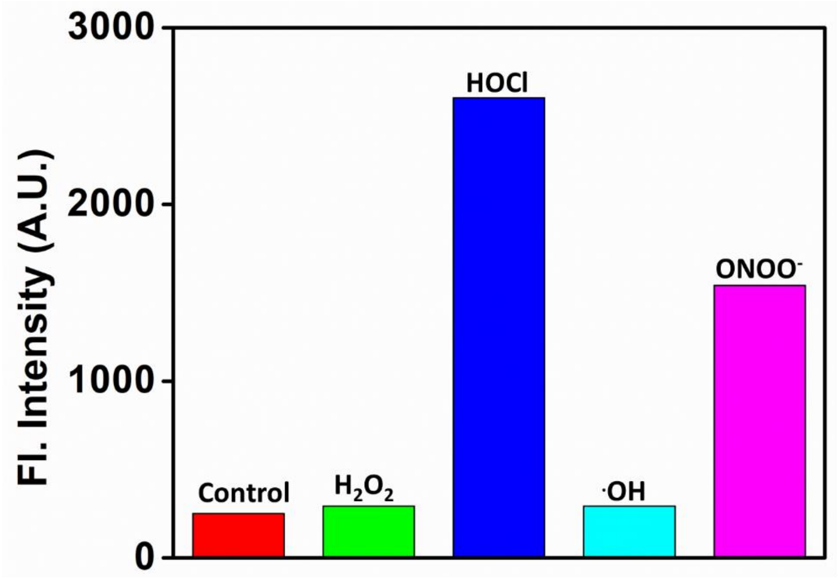

Figure S1. The selectivity of **AB** with different ROS was measured in a microplate reader.

5. **Time-dependent UV Study in the presence of H_2O_2**

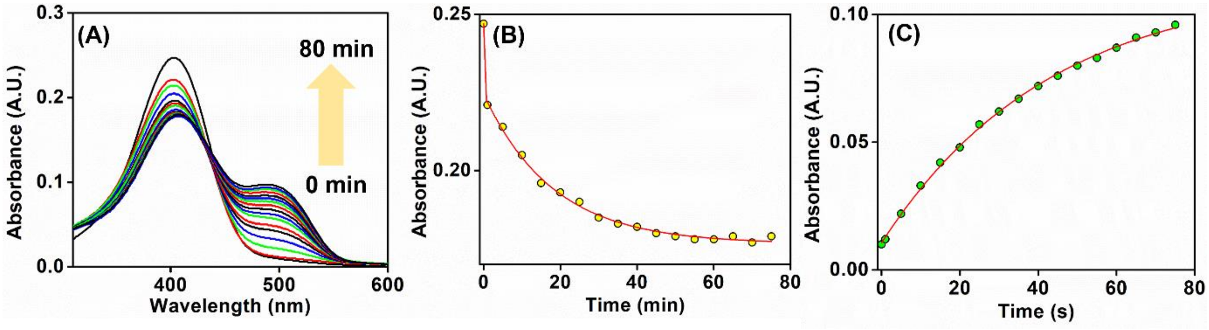

Figure S2. (A) Absorbance spectra of **AB** in the presence of 100 μM H_2O_2 at different time points up to 80 mins. (B) Decrease of absorbance peak around 400 nm and (C) increase of absorbance peak around 490 with time. Probe concentration: 10 μM.

6. **Time-dependent UV Study in the presence of HOCl**

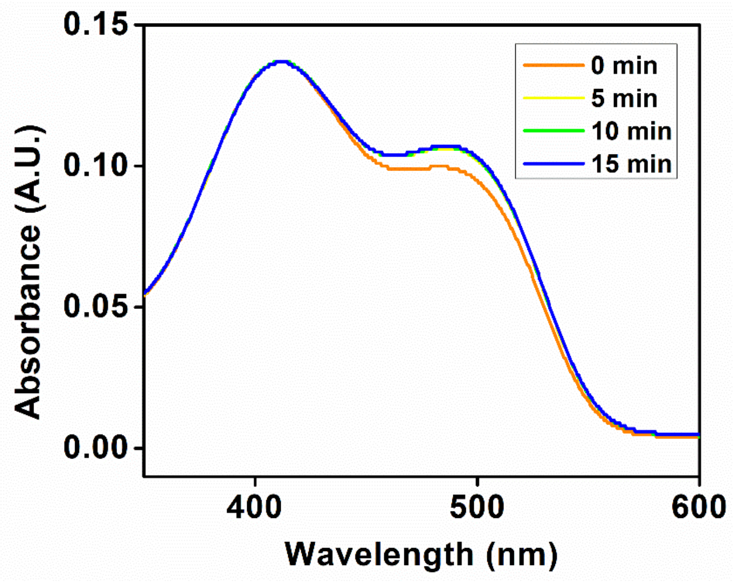

Figure S3. Absorbance spectra of **AB** in the presence of 100 μM HOCl at different time points up to 15 mins. Probe concentration: 10 μM.

7. Concentration Dependent emission study with H_2O_2

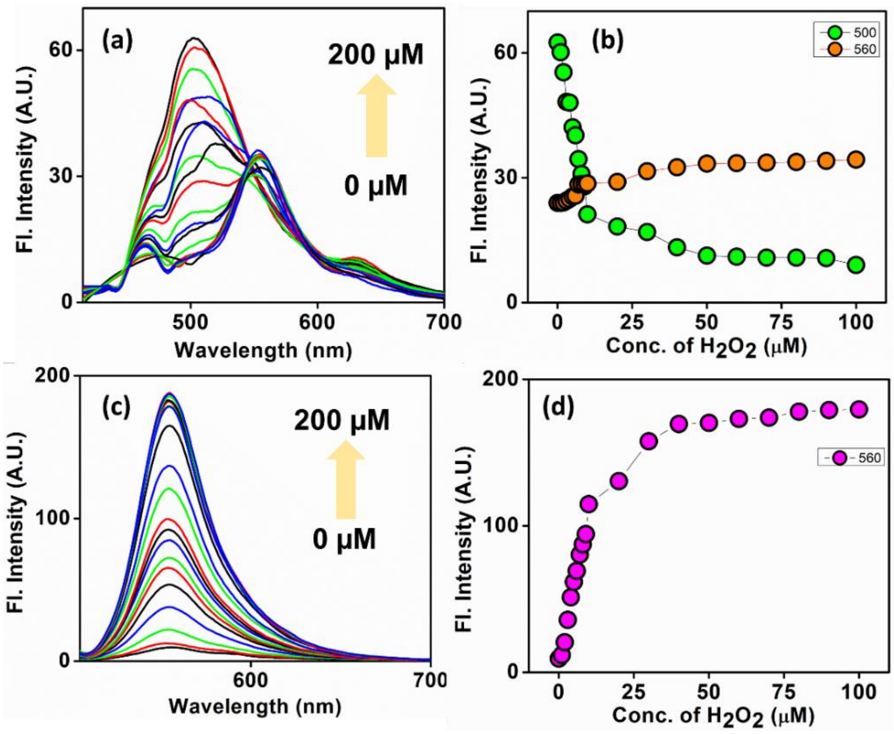

Figure S4. Emission spectra of **AB** in the presence of different concentrations of H_2O_2 (0–200 μM) in PBS (10 mM, pH 7.4, containing 0.1% DMSO), (a) λ_{ex} = 400 nm, (c) λ_{ex} = 490 nm; and their corresponding secondary plots (b,d). The H_2O_2 concentrations used were 100nm, 300 nm, 500 nm, 700 nm, 1-10 μM (10 points), 10-100 μM (10 points), and 200 μM.

8. pH dependent and Viscosity study

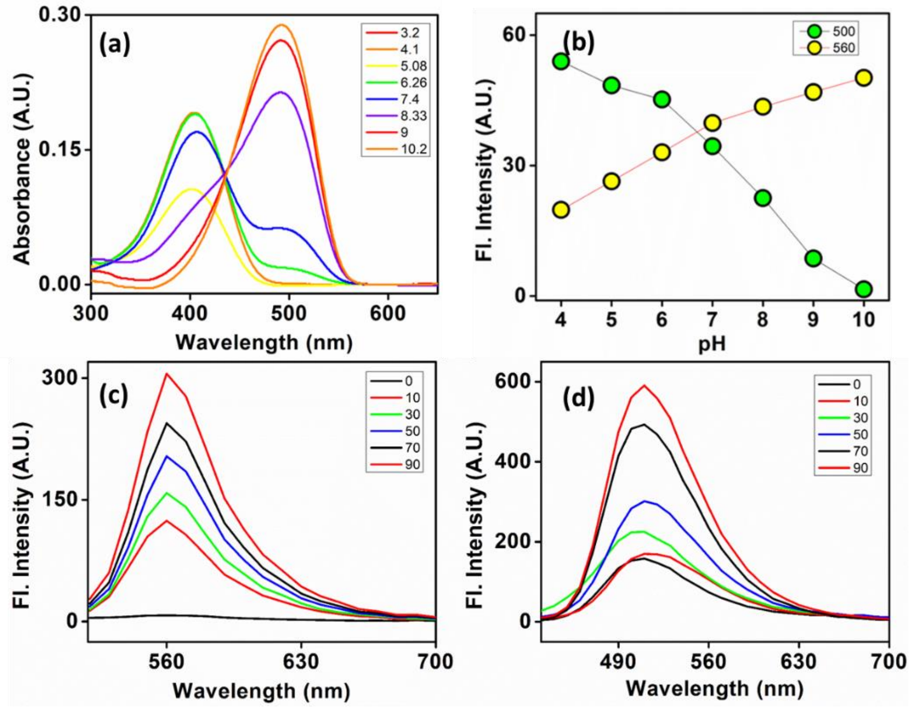

Figure S5. Absorbance spectra of **AB-OH** in the presence of different pH solutions from pH 3-10 and (b) their corresponding secondary plots. (c) Change in emission spectra of **AB** in different viscous solutions of 0-90% glycerol in water λ_{ex} = 400 nm, (d) Change in emission spectra of **AB-OH** in different viscous solutions of 0-90% glycerol in water λ_{ex} = 490 nm.

9. Time-dependent fluorescence Study in the presence of H_2O_2 and HOCl

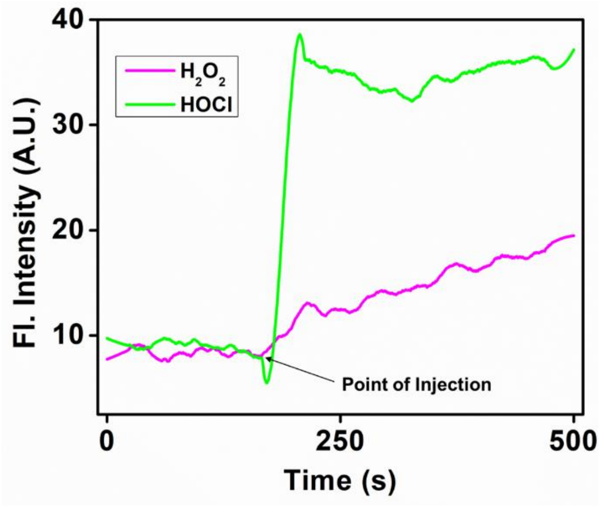

Figure S6. Fluorescence spectra of **AB** in the presence of 100 μM H_2O_2 and HOCl at different time points up to 500 seconds. Probe concentration: 10 μM.

10. HPLC Study in the presence of ROS

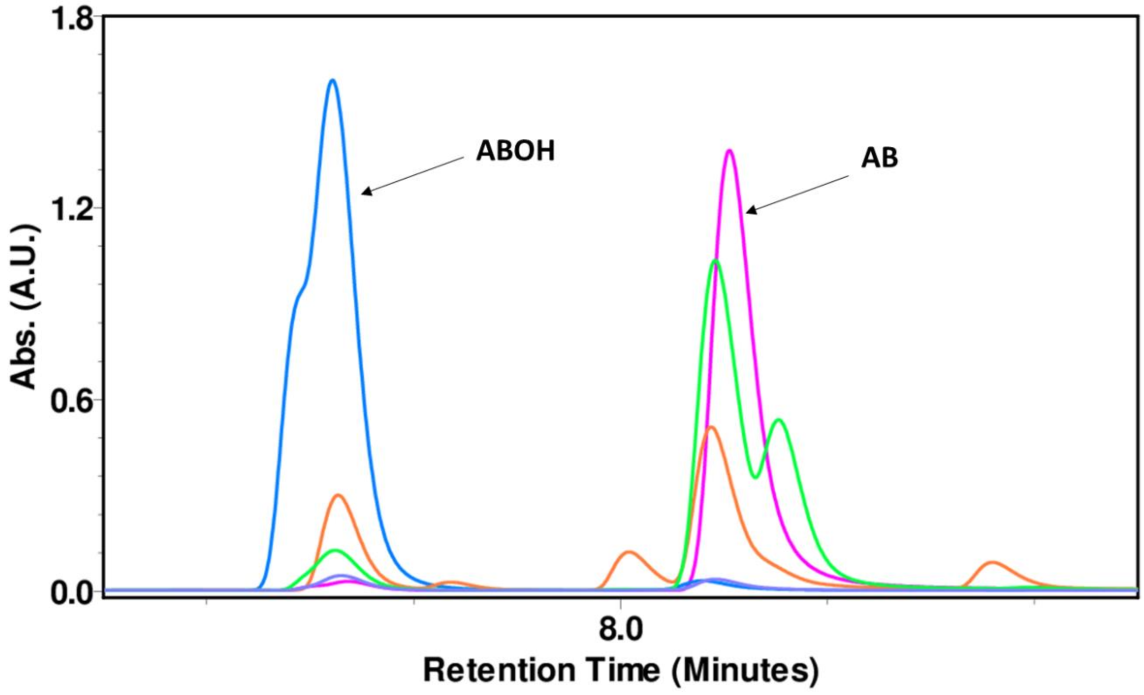

Figure S7. HPLC Chromatogram in the presence of KO_2 (Violet), H_2O_2 (Green), and HOCl (Orange) with AB (Pink) and AB-OH (Blue) as control. The absorbance value at 490nm is recorded against retention time.

The method used for the above HPLC study is described below:

Pump Mode: Gradient		Max Pressure: 4000 psi		
Time (min)	Flow	%A (Water)	%B (ACN)	Curve
	0.30	100	0	
10	0.30	0	100	6
12	0.30	0	100	6
13	0.30	100	0	6
15	0.30	100	0	6

11. Selectivity Study

The selectivity study of AB towards relevant bio-analytes was done in PBS buffer (10Mm, pH 7.4). The spectra were recorded after 30 minutes after the addition of the desired analytes.

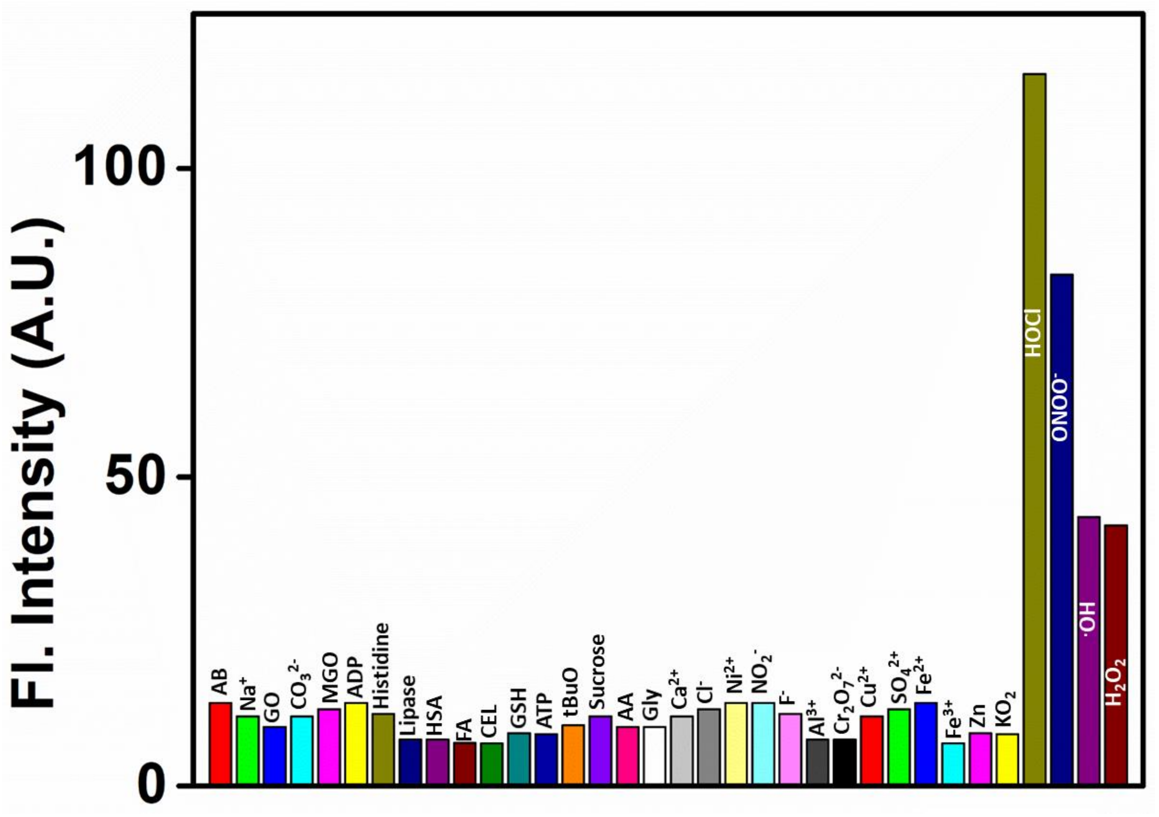

Figure S8. Selectivity study of AB with biologically relevant analytes.

12. MTT

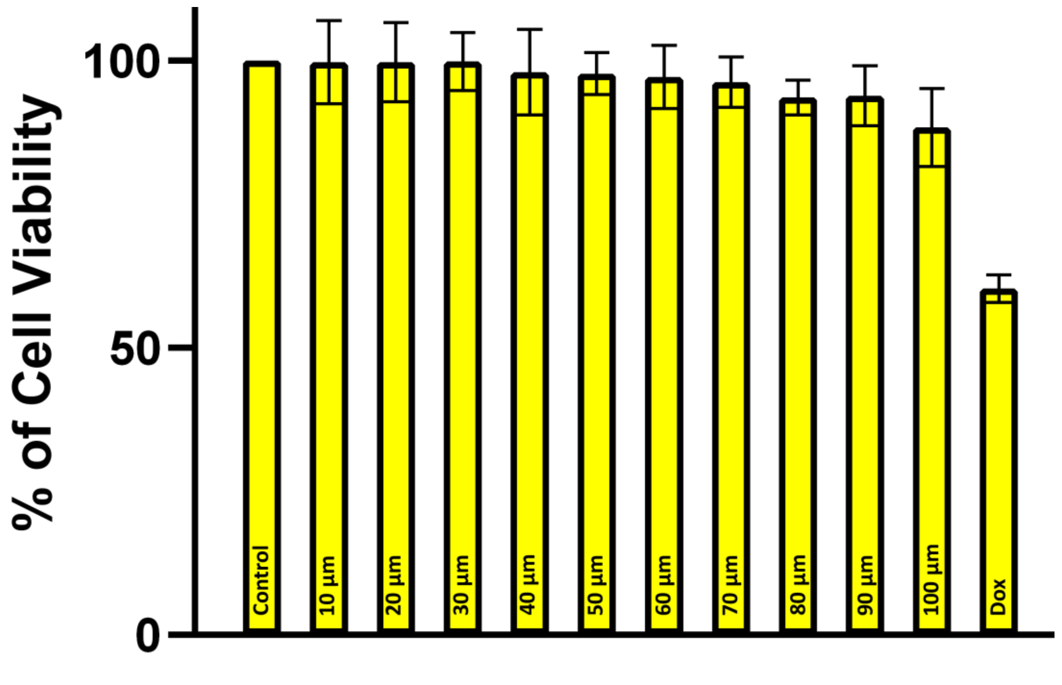

Figure S9. Cytotoxicity study using MTT Assay taking doxorubicin as a control with different concentrations of AB.

13. Mass spectrometric data

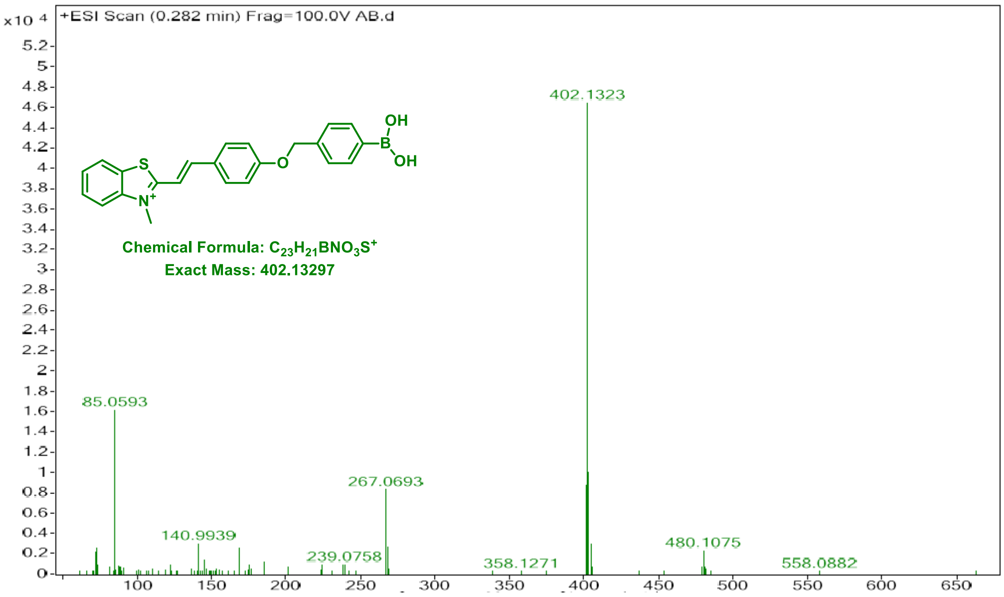

Figure S10. ESI-HRMS study of **AB.**

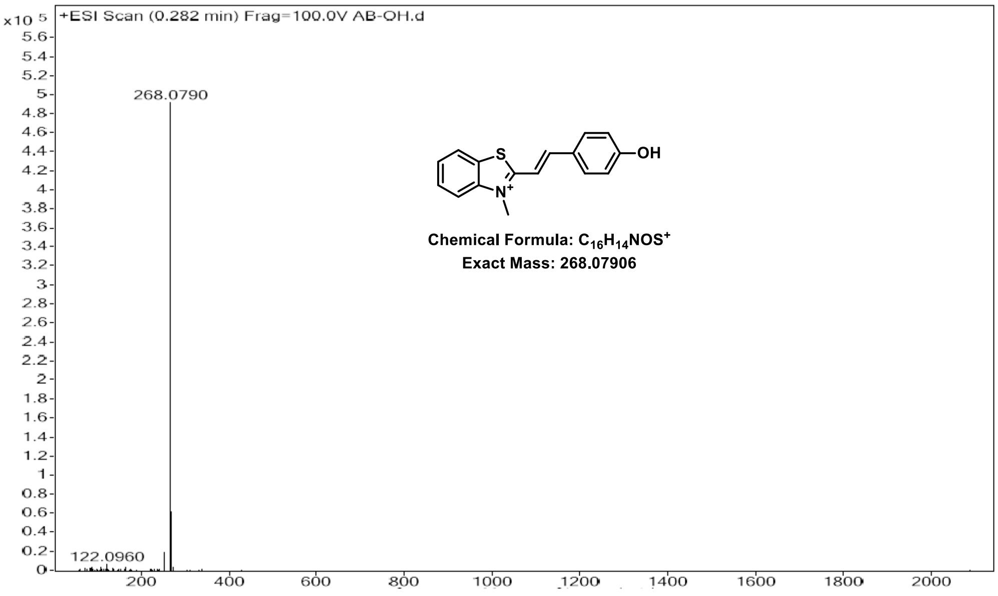

Figure S11. ESI-HRMS study of **AB-OH.**

14. Nuclear Magnetic Resonance (NMR) spectroscopy

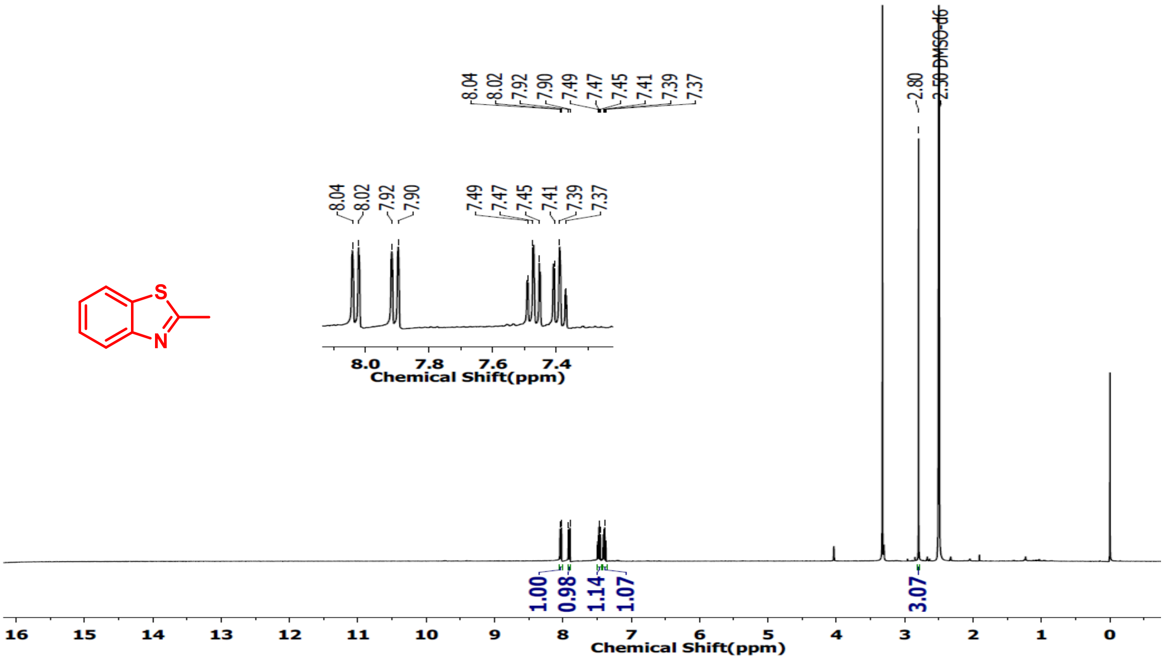

Figure S12. ^{1}H NMR of (A).

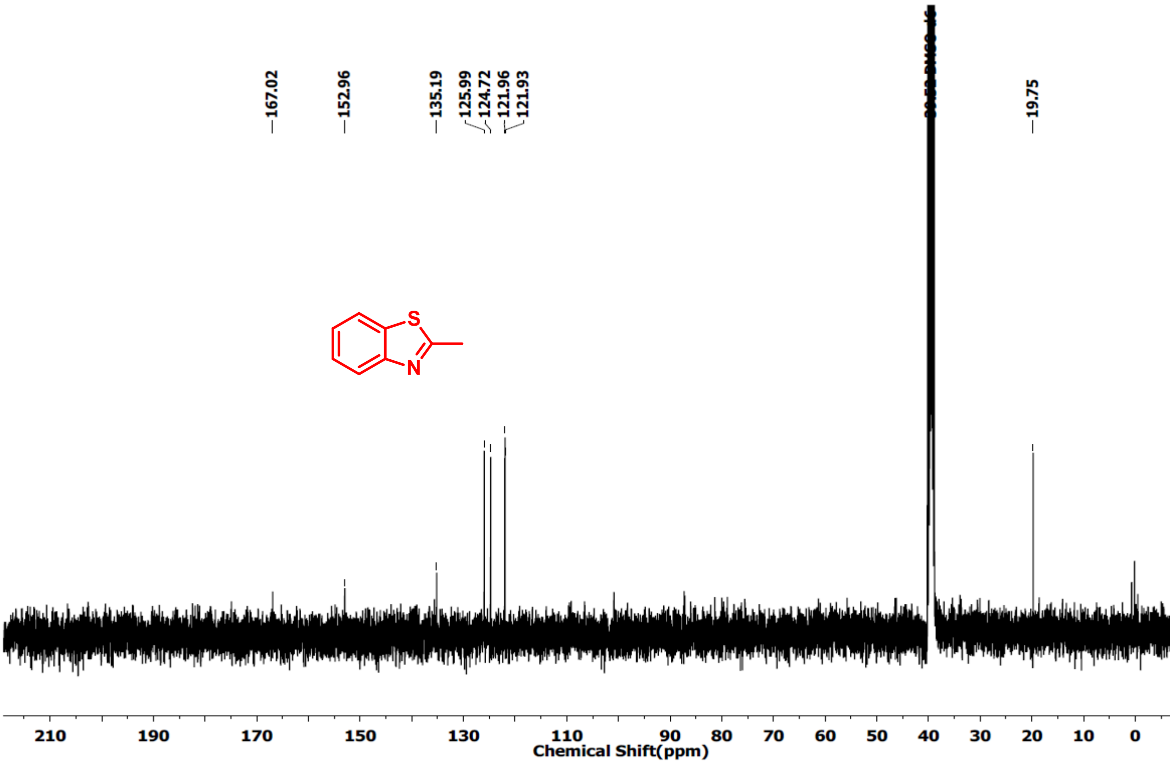

Figure S13. ^{13}C NMR of (A)

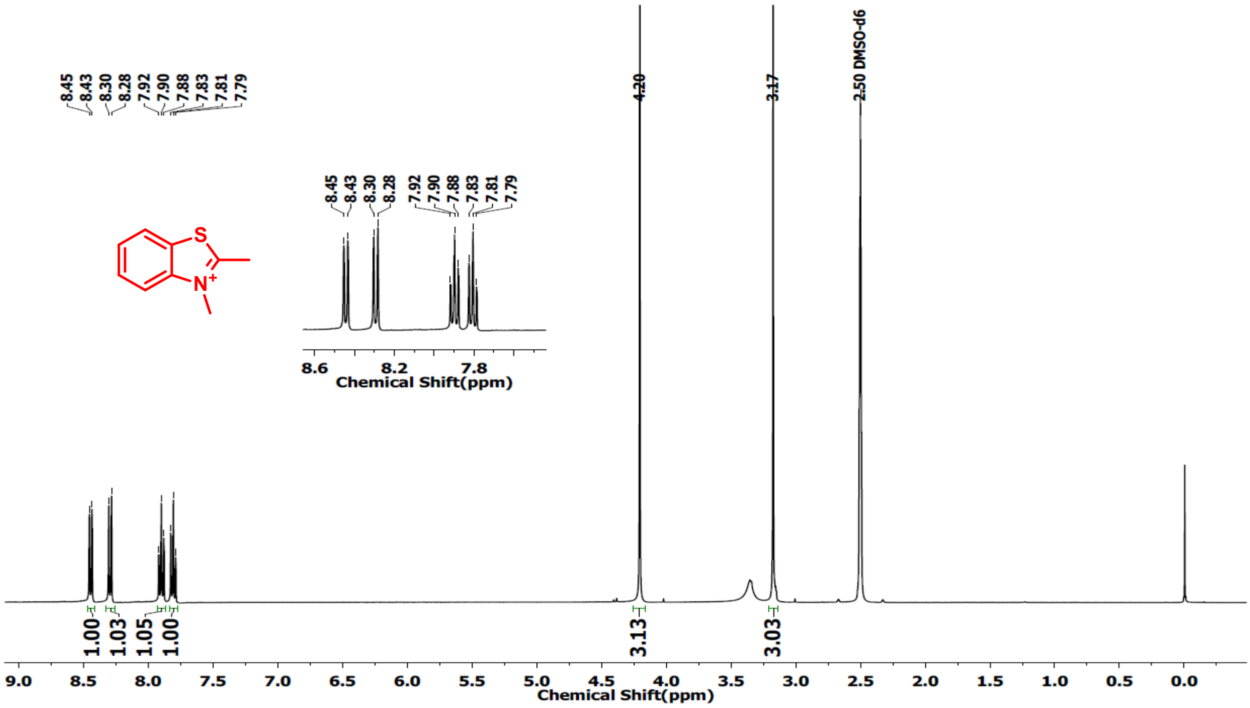

Figure S14. ^{1}H NMR of (B).

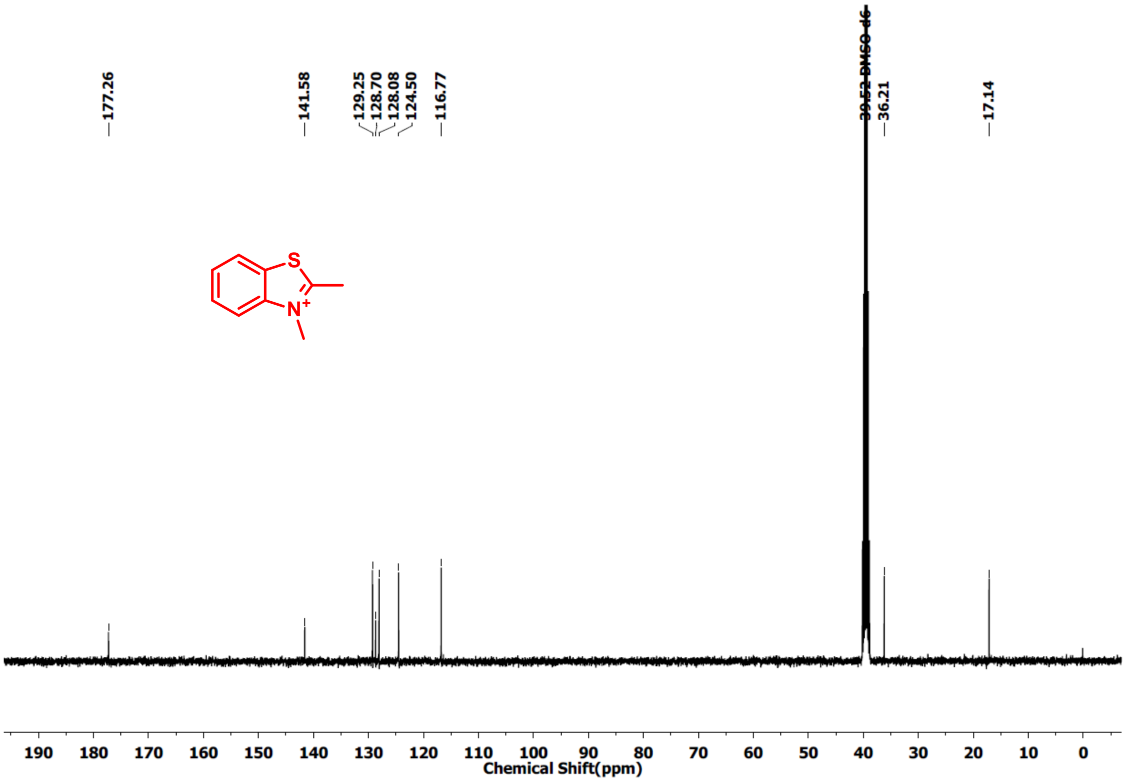

Figure S15. ^{13}C NMR of (B)

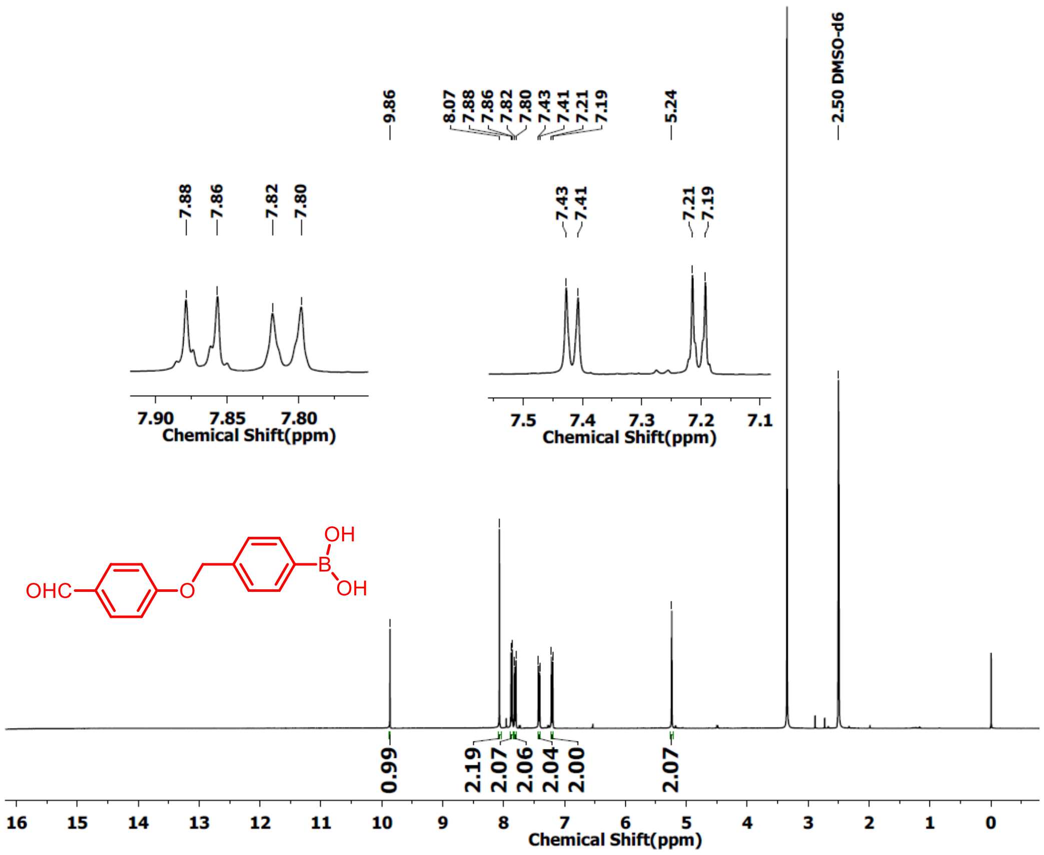

Figure S16. ^{1}H NMR of (C).

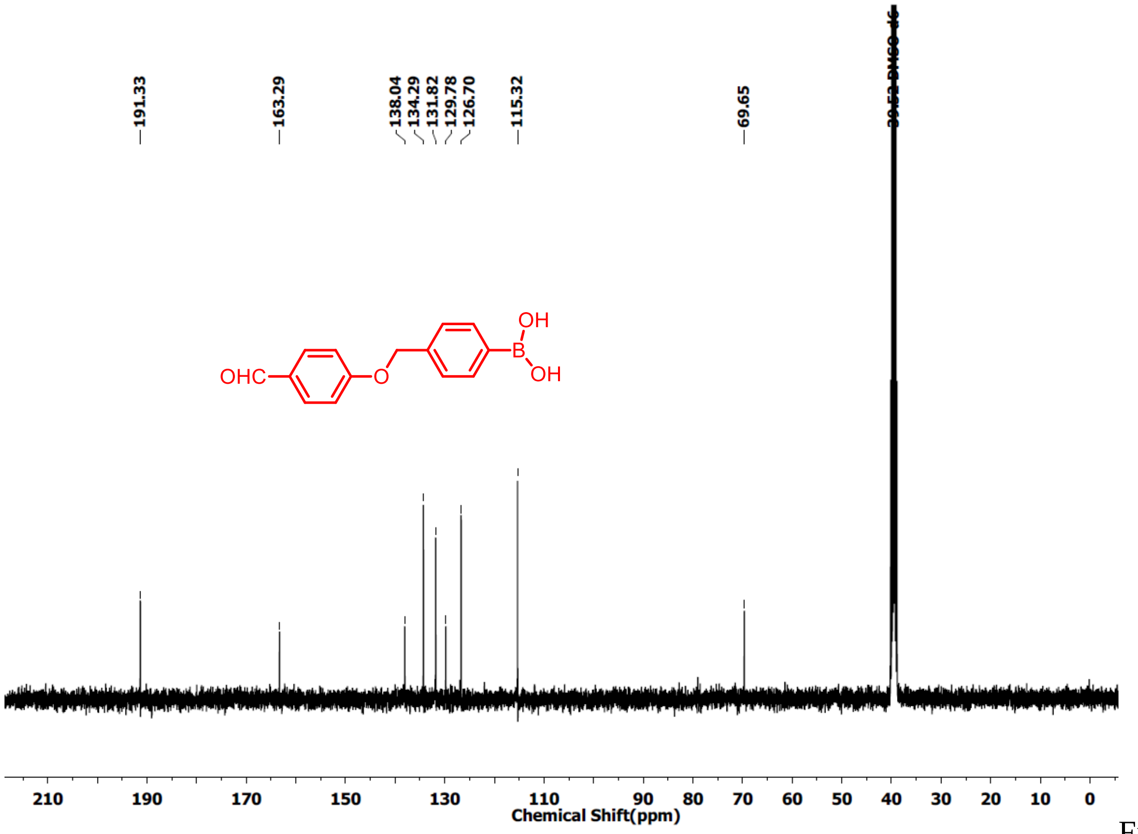

Figure

Figure S17. ^{13}C NMR of (C).

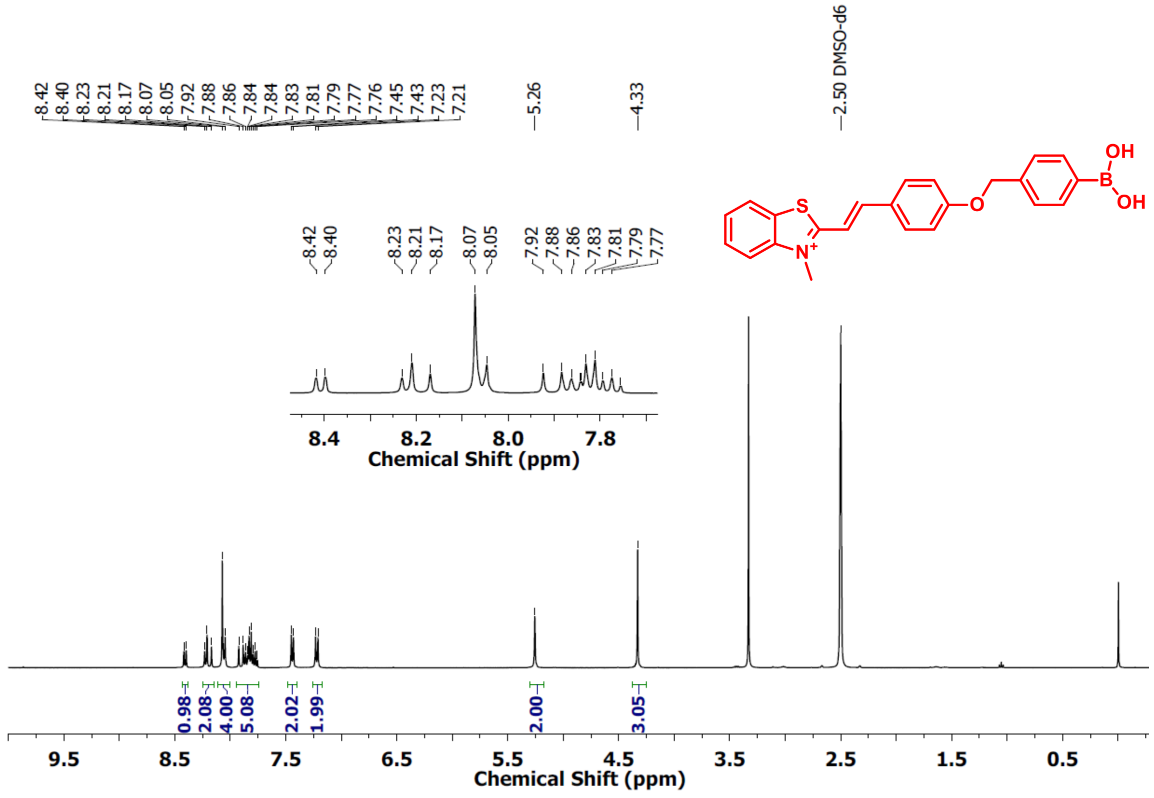

Figure S18. ^{1}H NMR of AB.

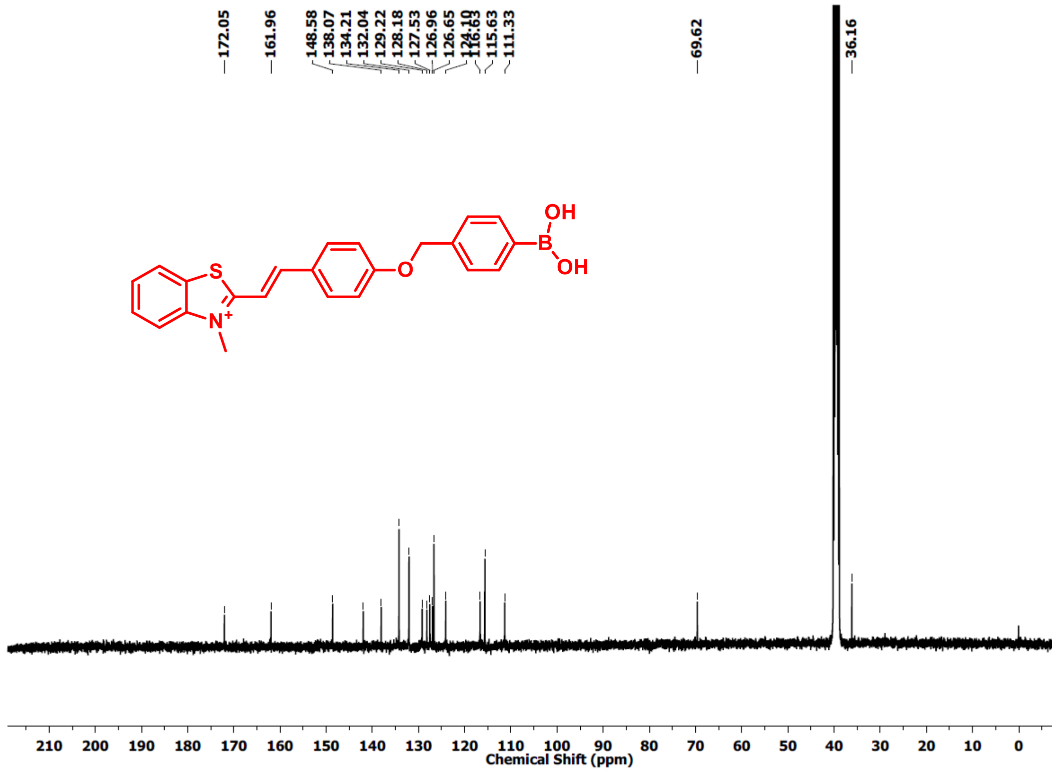

Figure S19. ^{13}C NMR of AB.

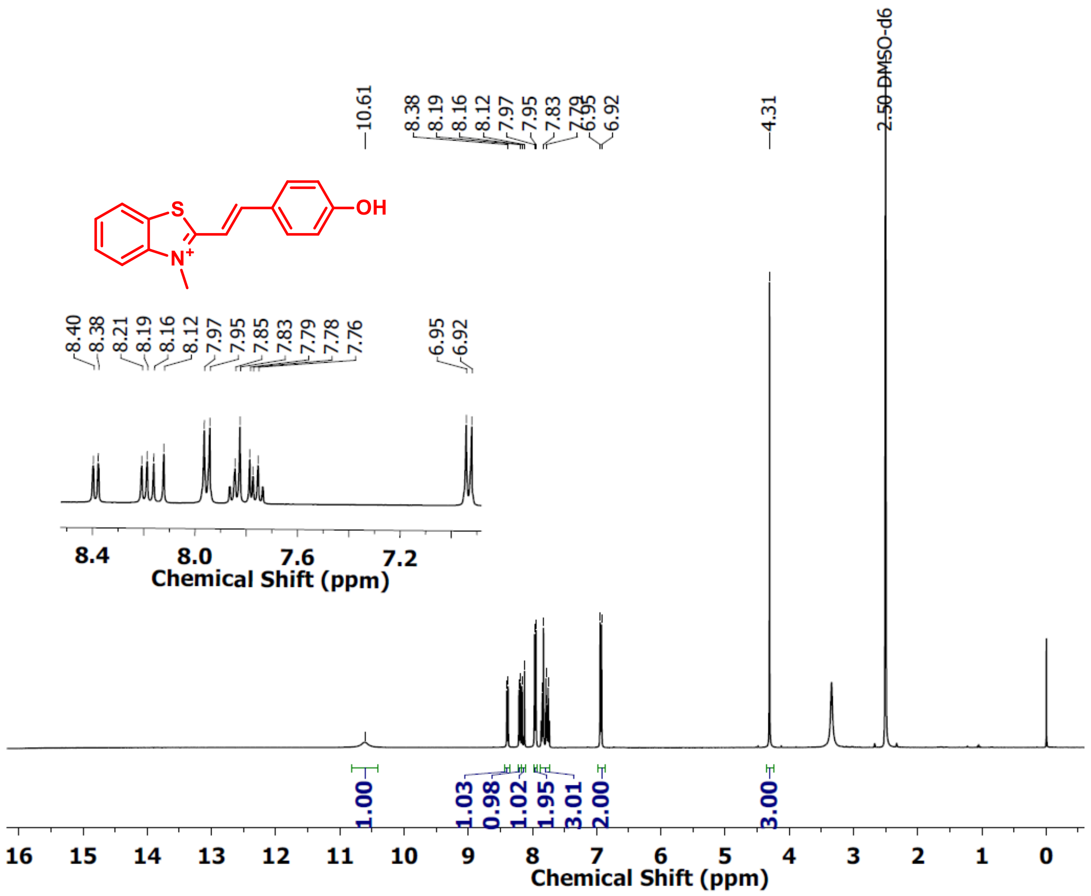

Figure S20. ^{1}H NMR of AB-OH.

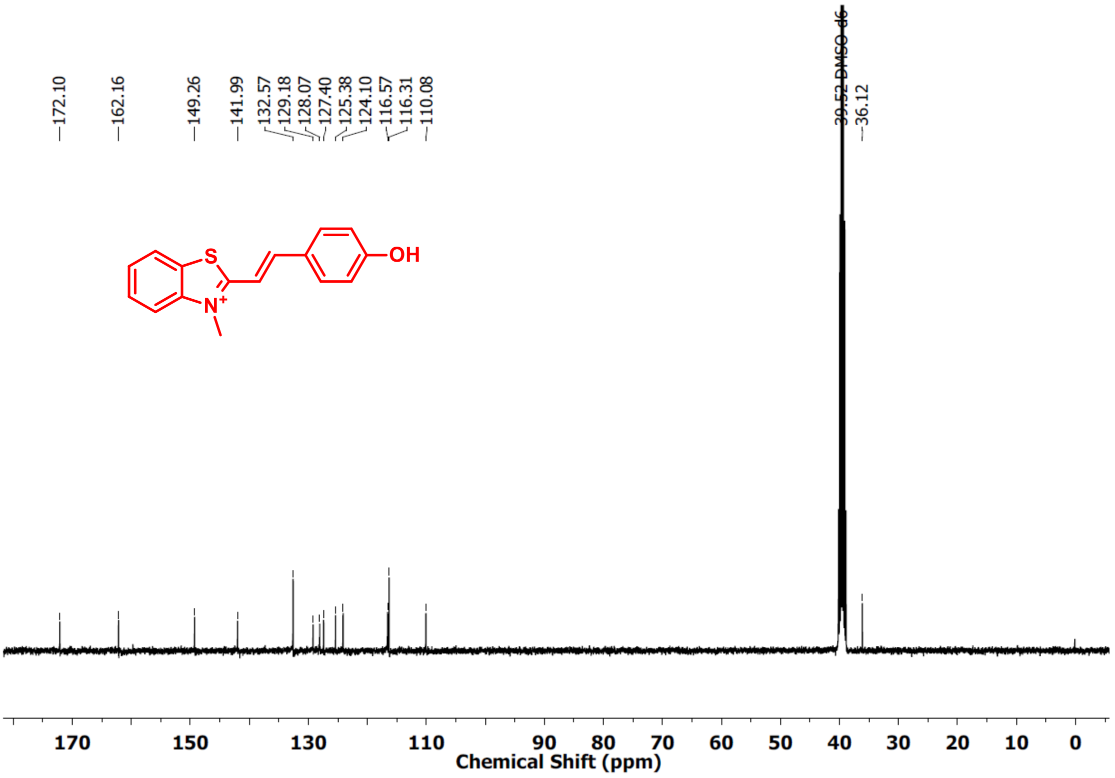

Figure S21. ^{13}C NMR of AB-OH.
